# Host Factors Required for Modulation of Phagosome Biogenesis and Proliferation of *Francisella tularensis* within the Cytosol

**DOI:** 10.1371/journal.pone.0011025

**Published:** 2010-06-11

**Authors:** Christine Akimana, Souhaila Al-Khodor, Yousef Abu Kwaik

**Affiliations:** 1 Department of Microbiology and Immunology, College of Medicine, University of Louisville, Louisville, Kentucky, United States of America; 2 Department of Biology, University of Louisville, Louisville, Kentucky, United States of America; Cairo University, Egypt

## Abstract

*Francisella tularensis* is a highly infectious facultative intracellular bacterium that can be transmitted between mammals by arthropod vectors. Similar to many other intracellular bacteria that replicate within the cytosol, such as *Listeria*, *Shigella*, *Burkholderia*, and *Rickettsia*, the virulence of *F. tularensis* depends on its ability to modulate biogenesis of its phagosome and to escape into the host cell cytosol where it proliferates. Recent studies have identified the *F. tularensis* genes required for modulation of phagosome biogenesis and escape into the host cell cytosol within human and arthropod-derived cells. However, the arthropod and mammalian host factors required for intracellular proliferation of *F. tularensis* are not known. We have utilized a forward genetic approach employing genome-wide RNAi screen in *Drosophila melanogaster-*derived cells. Screening a library of ∼21,300 RNAi, we have identified at least 186 host factors required for intracellular bacterial proliferation. We silenced twelve mammalian homologues by RNAi in HEK293T cells and identified three conserved factors, the PI4 kinase PI4KCA, the ubiquitin hydrolase USP22, and the ubiquitin ligase CDC27, which are also required for replication in human cells. The PI4KCA and USP22 mammalian factors are not required for modulation of phagosome biogenesis or phagosomal escape but are required for proliferation within the cytosol. In contrast, the CDC27 ubiquitin ligase is required for evading lysosomal fusion and for phagosomal escape into the cytosol. Although *F. tularensis* interacts with the autophagy pathway during late stages of proliferation in mouse macrophages, this does not occur in human cells. Our data suggest that *F. tularensis* utilizes host ubiquitin turnover in distinct mechanisms during the phagosomal and cytosolic phases and phosphoinositide metabolism is essential for cytosolic proliferation of *F. tularensis*. Our data will facilitate deciphering molecular ecology, patho-adaptation of *F. tularensis* to the arthropod vector and its role in bacterial ecology and patho-evolution to infect mammals.

## Introduction

*Francisella tularensis* is a Gram-negative facultative intracellular zoonotic bacterium that infects a broad range of small animals and causes tularemia [1], [2]. *F. tularensis* is classified into four closely related subspecies: *tularensis*, *holarctica*, *mediasiatica* and *novicida* [1], [2], [3], [4]. Despite its attenuation in humans, subspecies *novicida* is trafficked and replicates within macrophages similar to the most virulent subspecies [1], [5], [6]

Humans become infected with *F. tularensis* through contact with infected animal tissues, ingestion of contaminated food or water, inhalation of contaminated aerosols, and by arthropods, such as ticks, flies, mosquitoes, deer fly, and horsefly [1], [7], where the organism is present in the feces and not in the saliva of the arthropod vector [8]. Although arthropod transmission of *F. tularensis* to humans remains a concern worldwide [9], very little is known about the interaction of *F. tularensis* with the arthropod vectors.

Upon transmission to humans, *F. tularensis* is engulfed by macrophages, where the *F. tularensis*-containing phagosome (FCP) matures into an acidified late endosome-like phagosome with limited fusion to the lysosomes [1], [10], [11], [12], [13], followed by rapid bacterial escape into the cytosol within 30-60 min post-infection [10], [12], [14], [15], [16]. Various genes within the *F. tularensis* pathogenicity island (FPI) [17], such as *iglC*, are required for modulation of phagosome biogenesis and bacterial escape into the cytosol [1], [11], [12], [18], [19]. In mouse bone marrow-derived macrophages (BMM), *F. tularensis* re-enter the endocytic compartment within ∼20hrs of infection, via an autophagy-like process [10]. It is not known whether interaction of *F. tularensis* with autophagy also occurs in human-derived cells.

Several arthropod vector models for *Francisella* such as *Galleria mellonella* [20], *D. melanogaster* [1], [21], and mosquitoes [6] have been explored. Within *D. melanogaster*-derived cells, the FCP matures to a late endosome-like phagosome followed by bacterial escape into the cytosol, similar to mammalian cells [1]. Recent studies have established the genetically tractable model system, *D. melanogaster*, as an arthropod vector model system for *F. tularensis* [1], [21]. *F. tularensis* proliferates in adult flies and in *D. melanogaster-* and mosquitoe–derived cells [1], [21].

At least 90 loci of *F. tularensis* are required for evasion of lysosomal fusion and bacterial escape into the cytosol, and 34 bacterial loci are required for proliferation in the cytosol of human macrophages [22]. There are conserved as well as host species-specific genes of *F. tularensis* required for phagosomal escape and intracellular proliferation in human macrophages and *D. melanogaster*-derived cells [22], [23]. Interestingly, about 50% of the bacterial loci required for replication in *D. melanogaster*-derived cells are also required for proliferation in adult fruit flies [23]. The FPI genes, as well as the *mglA* global regulator, are required for modulation of phagosome biogenesis and escape into the cytosol of human-derived and *D. melanogaster*-derived cells [22], [23]. Therefore, some *F. tularensis* factors required for virulence in mammals are also required for virulence in the arthropod model system, but there are distinct molecular differences utilized by *F. tularensis* to exploit the two hosts [22], [23].

*D. melanogaster* S2 cells are macrophage-like cells that have been exploited to identify host factors that interact with several important pathogens [24], [25], [26], [27], [28], [29], [30], [31]. Since no arthropod or mammalian host factors are known to be required for intracellular growth of *F. tularensis*, we performed a genome-wide *D. melanogaster* RNAi screen to identify arthropod factors required for intracellular proliferation of *Francisella*. Our data show that 186 RNAi targets suppressed intracellular proliferation by *F. tularensis*. We silenced 12 mammalian homologues by RNAi and identified nine arthropod-specific factors and three common host species factors, PI4KCA, USP22, and CDC27 that were also required for replication in human cells. The PI4KCA and USP22 factors are required for cytosolic proliferation, while CDC27 is required for evasion of lysosomal fusion and phagosomal escape. Our approach has identified arthropod vector-specific factors as well as a few conserved mammalian factors required for modulation of phagosome biogenesis and bacterial escape into cytosol by *F. tularensis*.

## Results

### *D. melanogaster* RNAi screen to identify host factors required for infection by *F. tularensis*

**Figure 1:**
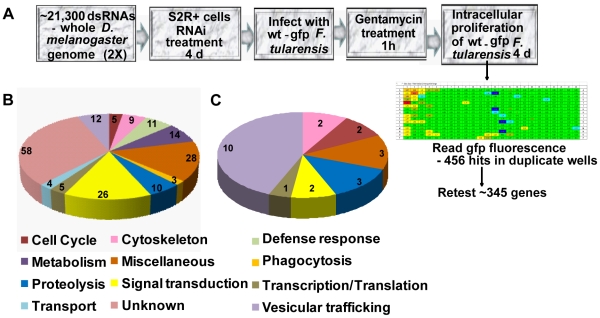
*D. melanogaster* RNAi screen and identification of host factors required for proliferation of *F. tularensis*. (A) Genome-wide screen design. Procedural outline for the screen including RNAi treatment, infection, and identification of hits from a z-score heat map. (B) Functional categories of RNAi targets that decreased intracellular proliferation of *F. tularensis* in the secondary screen and (C) functional categories of targets that were also identified to be required for intracellular proliferation of *L. monocytogenes*, *Mycobacterium*, or *Chlamydia* in the secondary screen. The number of host factors within each functional category in panels B and C are shown.

**Figure 2:**
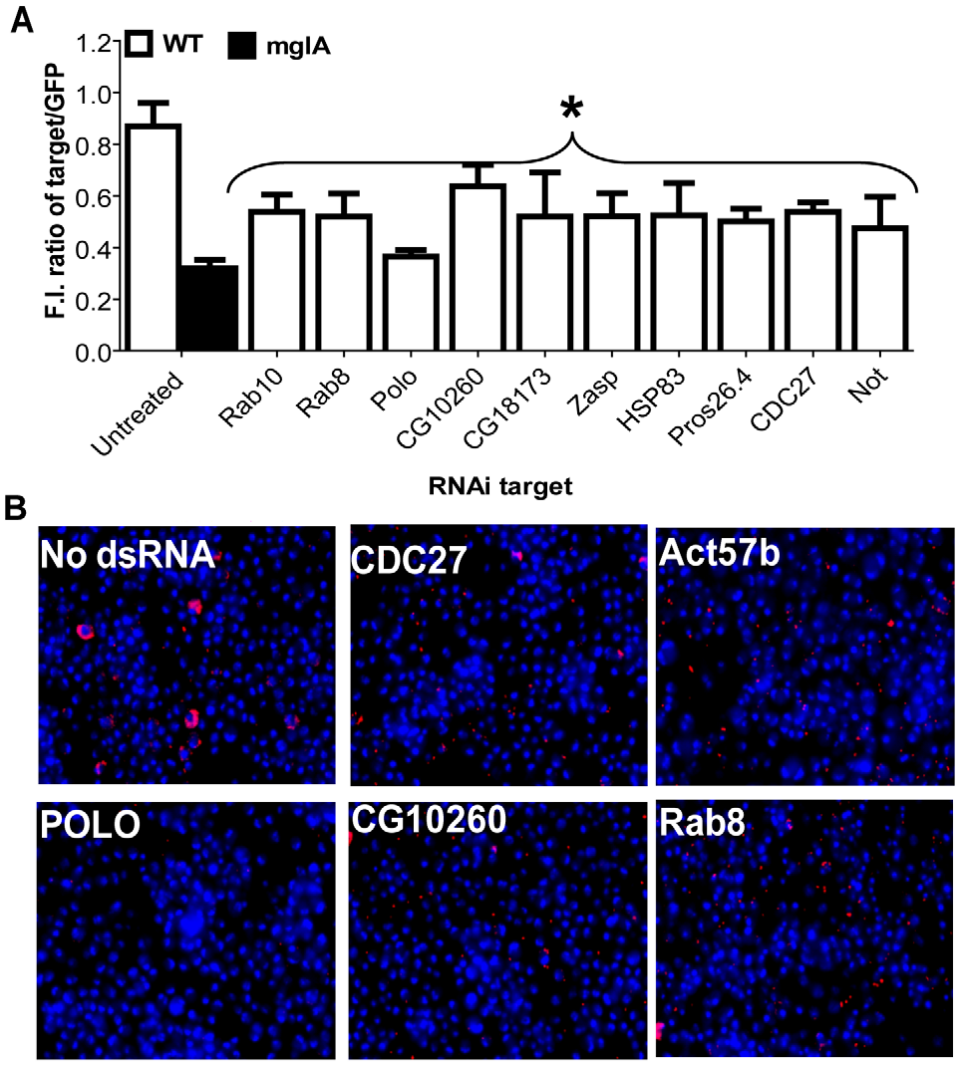
Representative host factors required for intracellular growth of *F. tularensis*. S2R+ cells were treated with RNAi targeting *gfp*, an arbitrary negative control, or the indicated representative RNAi and infected with *F. tularensis* expressing GFP. (A) At 4 days post-infection, the fluorescence intensity (F.I.) of *F. tularensis* GFP was measured. The GFP-expressing *mglA* mutant that is severely defective in intracellular proliferation was used as a control. Results of representative genes were obtained in two independent experiments, each with up to 4 wells. Asterisk indicates RNAi that produced a statistically significant decrease in *F. tularensis* GFP fluorescence intensity versus control RNAi, as described in materials and methods. Error bars represent standard error of the mean. (B) Representative RNAi-treated cells were fixed and stained with Hoechst dye (blue) and bacteria were labeled with antibodies (red) at 24 h post-infection.

There are conserved as well as host species-specific genes of *F. tularensis* required for phagosomal escape and intracellular proliferation in human macrophages and *D. melanogaster*-derived cells [22], [23]. To identify arthropod host factors involved in intracellular proliferation of *F. tularensis*, we developed a GFP fluorescence plate reader-based high throughput RNAi screen in *D. melanogaster* –derived cells (Fig. 1A). For the high throughput primary genome-wide screen, ∼21,300 genes were targeted by RNAi in S2R+ cells that were infected with GFP-expressing *F. tularensis* for 2 h at MOI of 10, followed by killing of extracellular bacteria by gentamicin. The intensity of GFP fluorescence was measured at 4 days post-infection. Duplicate plates were processed side by side and RNAi targets that suppressed intracellular bacterial proliferation were considered hits if similar results were obtained in both plates (Fig. 1A). To score the strength of suppression of intracellular bacterial proliferation (down phenotype), the Z-scores below −2, −1.2, and −0.75 were considered as strong, medium, and weak effects, respectively (See experimental procedures). To score the strength of enhanced intracellular bacterial proliferation (up phenotype), Z-scores above 3 and 2 were considered as strong and medium effects, respectively (see experimental procedures). Internal controls in each plate included thread, which is an RNAi target that affects cell viability. Another internal control targeting an arbitrary *gfp* (not related to bacteria or cells) was included to normalize all raw data (Fig. 2). Overall, 456 RNAi targets that affected intracellular proliferation of *F. tularensis* were identified in the primary screen (data not shown). We re-tested the identified targets after exclusion of genes that affect host viability and transcription/translational processes [32]. We also included a list of 23 RNAi targets (see materials and methods) not identified in our primary screen, but which have been shown in other *D. melanogaster* RNAi screens to suppress the infection by *Mycobacterium*, *Chlamydia*, and *L. monocytogenes* [26], [29], [30]. Overall, 345 RNAi targets were retested at least 4 times, and a total of at least 186 RNAi targets suppressed intracellular proliferation of *F. tularensis*, consistently and reproducibly (Table S1, down phenotype), while 20 RNAi species were confirmed to enhance intracellular proliferation of *F. tularensis* (Table S1, up phenotype). Using trypan blue exclusion, we confirmed that all the RNAi targets that suppressed or enhanced intracellular proliferation in the secondary screen did not affect host cell viability. The 186 genes confirmed in the secondary screen to suppress intracellular proliferation were classified into 12 functional categories, predicted according to their annotation on the flybase website (Table S1). These include unknown (58 genes; 31%), transcription/translation (5 genes; 3%), miscellaneous (28 genes, 15%), metabolism (14 genes; 8%), signal transduction (26 genes; 14%), proteolysis (10 genes; 5%), cytoskeleton (9 genes; 5%), cell cycle (5 genes; 3%), defense response (11 genes; 6%), transport (4 genes; 2%), vesicular trafficking (12 genes; 6%), and phagocytosis (3 genes; 2%) (Fig 1B, Table S1). Only RNAi targets that affected proliferation of *F. tularensis* in at least 3 out of 4 independent experiments are shown (Fig 1B, Table S1). Immunofluorescence examination of the infection of the RNAi-treated cells confirmed suppression of intracellular proliferation, compared to the GFP control (Fig. 2B). Comparing our screen to the *L. monocytogenes* [30], *Mycobacterium* [26], and *Chlamydia* [29] screens, we identified 23 genes that were required for the intracellular infection of other intracellular pathogens (Fig. 1C and Table S1), indicating that few common host targets are required for infection by other intracellular pathogens. Interestingly, 10 of these common host genes were involved in vesicular trafficking, which is consistent with the prediction that various intracellular pathogens may utilize some conserved mechanisms of manipulating the vesicular trafficking pathways.

### Mammalian host factors required for intracellular replication of *F. tularensi*s

**Figure 3:**
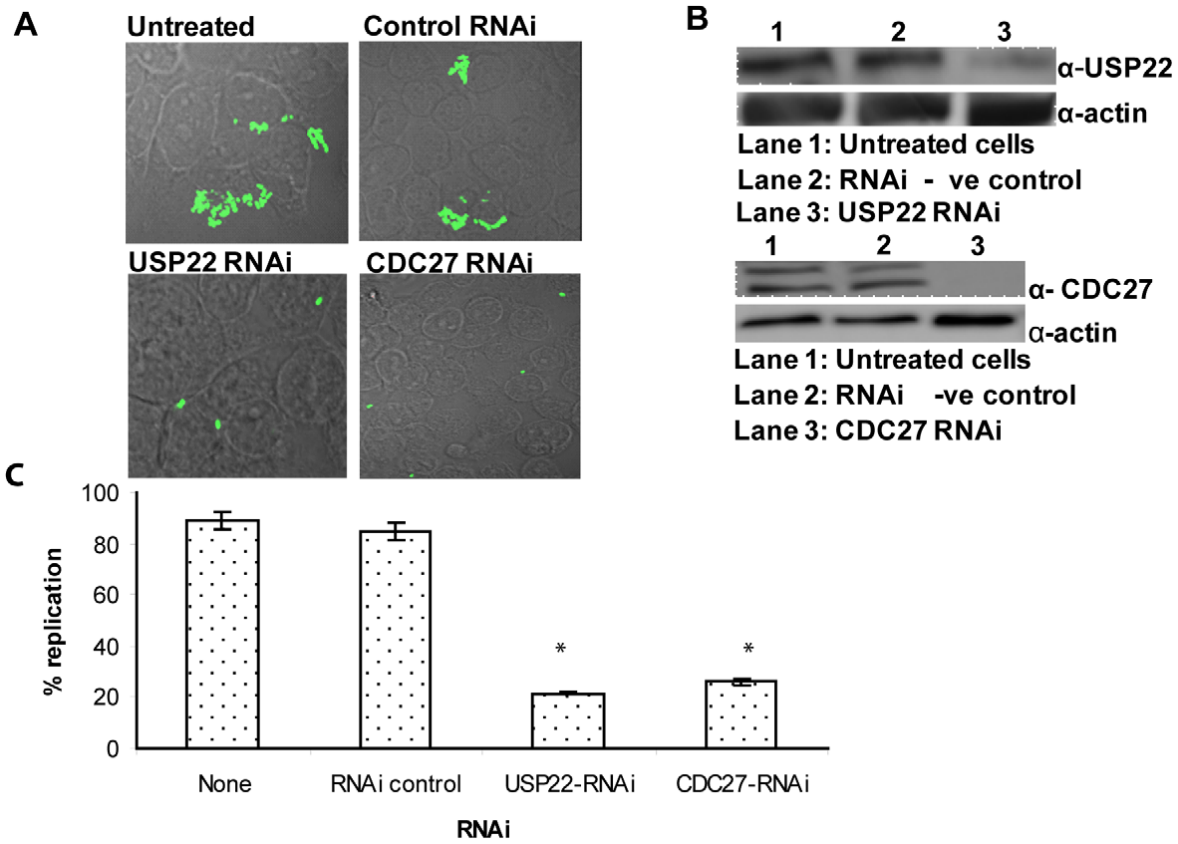
Suppression of intracellular replication of *F. tularensis* within CDC27 and USP22 RNAi-silenced HEK293T cells. A. Representative microscopy images of HEK293T cells at 8 h after infection by *F. tularensis*. HEK293T cells were either left untreated or treated with the RNAi negative control or the corresponding RNAi for USP22 or CDC27 for 48 h before infection, then infected with MOI of 10 by the WT strain U112 for 1 hr, followed with 1 hr gentamicin and additional 6 h of incubation for a total of 8 h. B. Western blot results confirming specific gene silencing. Membranes were blotted with specific antibodies against USP22 or CDC27, then stripped and re-probed with the anti-actin antibody as a loading control. C. Effect of USP22 and CDC27 silencing on bacterial replication after 8 h of infection. HEK293T cells were either left untreated or RNAi treated for 48 h before infection, then infected with the WT strain U112. We considered cells harboring 6–15 bacteria after 8 h of infection as normal WT levels of replication. At least 100 infected cells from different field were analyzed in each experiment. Data are the results of one experiment representative of three independent experiments. The asterisk indicates statistically significant differences between the control and the USP22 and CDC27 RNAi- treated cells.

**Figure 4:**
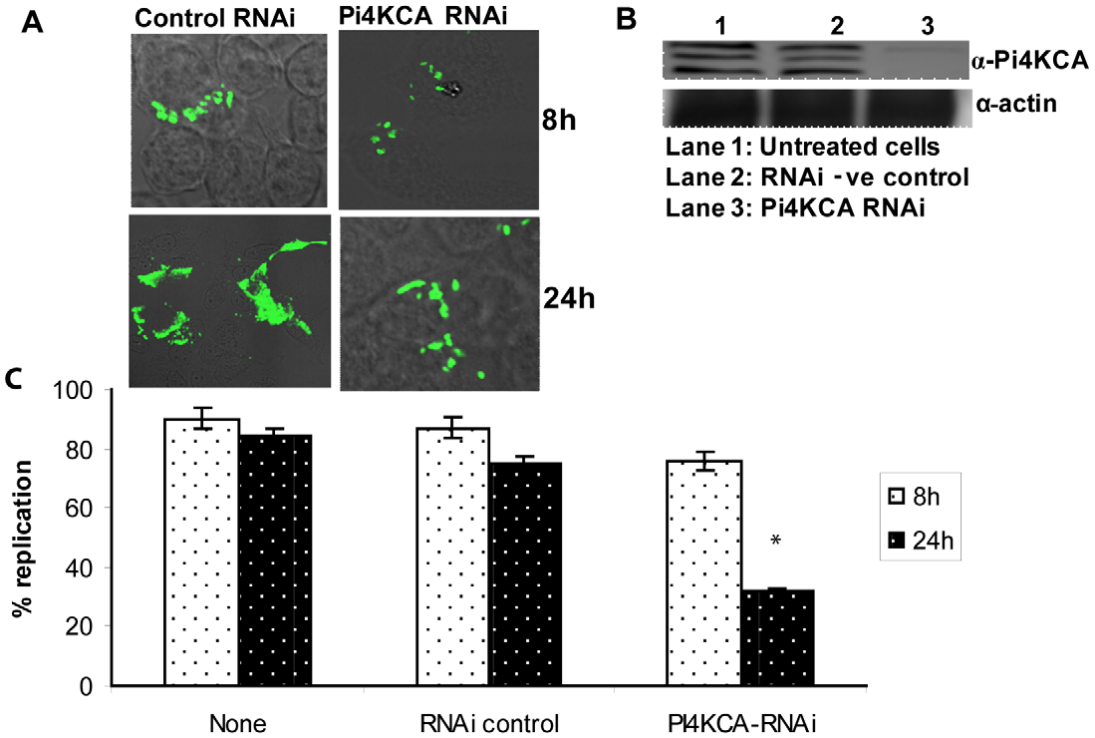
Late cessation of intracellular proliferation within PI4KCA-silenced cells. A) Representative microscopy images of HEK293T cells after 8 and 24 h infection by *F. tularensis*. HEK293T cells were either treated with the RNAi negative control or the PI4KCA RNAi for 48 h before infection, then infected with MOI of 10 with the WT strain U112 for 1 hr, followed by 1 h gentamicin and an additional 6 h (left panel) or 22 h (right panel) of incubation. B. Western blot results confirming PI4KCA gene silencing. Membranes were blotted with anti-PI4KCA antibody, then stripped and re-probed with the anti-actin antibody as our loading control. C. Effect of PI4KCA silencing on bacterial replication after 24 h of infection. HEK293T cells were either left untreated or RNAi treated for 48 h before infection, then infected with the WT strain. Depending on the MOI that we used, we considered cells harboring 6–15 bacteria after 8 h of infection and more than 25 bacteria after 24 h to reflect the normal WT levels of replication. At least 100 infected cells from different fields were analyzed in each experiment. Data are the results of one experiment representative of three independent experiments. The asterisk indicates statistically significant difference between the control and the PI4KCA RNAi- treated cells.

Although we predicted that the majority of the identified arthropod factors to be host species-specific, we hypothesized that few of the human homologues of the arthropod host factors may also be necessary for the intracellular replication of *F. tularensis* within human cells. Therefore, from the primary and secondary RNAi screens, we selected a group of 12 genes encoding proteins with different functional categories to be silenced in human cells. Six of the selected genes were identified in the primary screen only, while the other 6 were identified in both screens. The selection criteria of this group of genes were based on the existence of human homologues and on the major role of these genes in intracellular bacterial replication within *D. melanogaster* cells in the primary and secondary screens (Table S1, marked by an *). Since *F. tularensis* infects both phagocytic and non-phagocytic cells [33], we performed silencing in HEK293T cells, due to the high level of efficiency for RNAi silencing in these cells. Importantly, we have recently shown that evasion of lysosomal fusion and escape of *F. tularensis* into the cytosol in HEK293T cells is indistinguishable from human monocytes-derived macrophages (Al-Khodor et al, unpublished data). Cell viability was determined by trypan blue exclusion, and none of the RNAi targets used affected viability compared to the negative RNAi control or mock-treated cells. Western blot analysis confirmed all the 12 RNAi target genes were effectively and specifically silenced within 48 h by the respective specific RNAi (Fig. 3B, 4B, and data not shown). The data showed that nine of the RNAi targets tested had no detectable effect on intracellular proliferation, despite their effective silencing by the specific RNAi used (data not shown). Silencing of mammalian ubiquitin-specific peptidase USP22, ubiquitin ligase CDC27, and the PI4 kinase PI4KCA had temporal and spatial effects on intracellular proliferation. The data showed that at 8 and 24 h post-infection (Fig. 3), specific silencing of USP22 and CDC27 inhibited bacterial replication throughout the intracellular infection period, compared to the negative control RNAi-treated or untreated cells (Fig. 3A). Depending on the MOI, we considered cells harboring 6–15 bacteria after 8 h of infection and more than 25 bacteria after 24 h to reflect the normal WT levels of replication. At 8 h post infection, only ∼20% of the USP22- and CDC27-silenced infected cells showed normal levels of replication. In contrast, the WT strain proliferated in most cells (∼90%) that were either untreated or treated with the RNAi negative control (Fig. 3C).

In contrast, silencing of PI4KCA in HEK293T cells did not affect intracellular replication of *F. tularensi*s by 8 h of infection. Indeed, ∼90% of the untreated or the RNAi control-treated cells infected with the WT strain harbored 6–15 bacteria by 8 h. Similarly 76% of the PI4KCA RNAi-treated cells harbored 6–15 bacteria per cell by 8 h. At 24 h post infection the WT strain in both untreated or RNAi control treated cells showed increased bacterial numbers in ∼80% of the infected cells (Fig. 4A, C). However, in the PI4KCA RNAi-treated cells, the bacterial numbers did not increase at 24 h compared to the 8 h time point. Our data indicated a temporal requirement of the PI4KCA and its role in PI4 metabolism in later stages of cytosolic proliferation of *F. tularensis*.

### The role of mammalian USP22, CDC27 and PI4KCA in phagosome biogenesis of *F. tularensi*s

The FCP transiently matures to an acidified late endosome-like phagosome for ∼30 min, followed by escape of the organisms into the cytosol by 30–60 min (see [1] for a recent review). While the *F. tularensis* factors required for modulation of phagosome biogenesis and escape into the cytosol of human-derived and *D. melanogaster*-derived cells have been recently identified [22], [23], the host factors involved in these processes are not known. We postulated that CDC27, USP22 and PI4KCA suppressed intracellular bacterial replication due to alteration in biogenesis of the FCP in the RNAi-silenced cells. To test this hypothesis, we examined intracellular trafficking of the WT strain within non treated or RNAi-treated HEK293T cells and used the *iglC* mutant, which is defective in evasion of lysosomal fusion, as a control.

**Figure 5:**
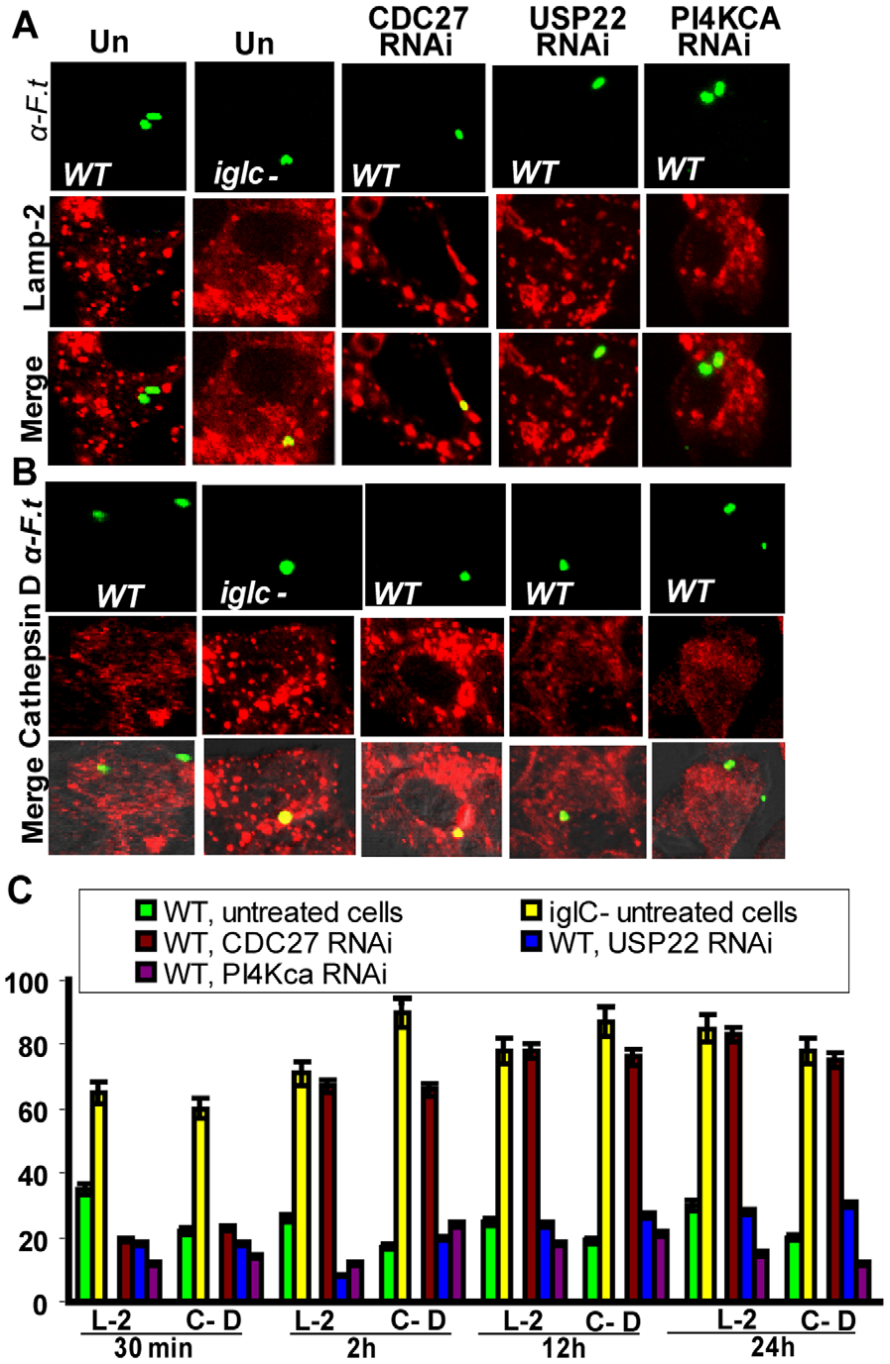
CDC27 but not USP22 or PI4KCA is required for modulation of phagosome biogenesis. A. Representative confocal microscopy images that show co-localization of the late endosomal/lysosomal marker LAMP-2 with the phagosomes harboring the *iglC* mutant in the untreated HEK293T cells (UN) as well as the WT strain in the RNAi-treated cells at 2 h after infection. B. Representative confocal microscopy images that show co-localization of the lysosomal enzyme cathepsin D with the phagosomes harboring the *iglC* mutant in the untreated cells (UN), as well as the WT strain in the RNAi-treated cells. C. Quantification of co-localization of the phagosomes with LAMP-2 (L-2) and cathepsin-D (C–D) markers at various time points. The results are based on examination of at least 100 bacteria at 2 h after infection analyzed by confocal microscopy in 2 independent experiments performed in triplicate and the error bars represent standard deviation.

We examined co-localization of the phagosomes with the late endosomal/lysosomal marker LAMP-2 and the lysosomal marker cathepsin D at 30 min and 2 h after infection (Fig. 5). As a negative control for LAMP-2 and cathepsin D co-localization we used the WT strain in the RNAi control-treated or non- treated cells. At 30 min after infection, ∼65% of the *iglC* mutant-containing phagosomes co-localized with LAMP-2 and cathepsin-D, as previously shown [34] (Fig. 5). No significant difference (Student *t*-test, *p*>0.1) in LAMP-2 andcCathepsin-D co-localization was observed between the WT strain-containing phagosomes in the untreated and the RNAi-treated cells (Fig. 5).

At 2 h after infection, only 26% of the WT strain-containing phagosomes co-localized with the late endosomal/lysosomal marker LAMP-2 (Fig. 5A and C), while 71% of the *iglC* mutant-containing phagosomes showed persistent co-localization with the LAMP-2 marker. Silencing of USP22 and PI4KCA did not affect co-localization with LAMP-2 when compared to untreated cells (Fig. 5A and C), indicating successful modulation of phagosome biogenesis. In contrast, 67% of the WT strain-containing phagosomes in the CDC27-silenced cells co-localized persistently with the LAMP-2 marker (Fig. 5A and C). This suggested a role for CDC27, but not USP22 or PI4KCA, in modulation of phagosome biogenesis by *F. tularensis*.

Similar results to co-localization of the FCP with LAMP-2 were observed with co-localization of the FCP with the lysosomal marker cathepsin-D (Fig 5B and C), where a small number of WT strain FCPs co-localized with this marker at 30 min in control cells. In contrast, 70–90% of the *iglC* mutant-containing phagosomes showed persistent acquisition of Cathepsin-D, indicating fusion with the lysosomal compartments. Silencing of USP22 and PI4KCA had no significant effect (Student *t*-test, *p*>0.2) on co-localization of the WT strain-containing phagosomes with cathepsin-D compared to non-treated cells (Fig. 5B and C). This indicates that evasion of lysosomal fusion by *F. tularensis* is independent of USP22 and PI4KCA. In contrast, the WT strain-containing phagosomes showed persistent co-localization with the lysosomal marker cathepsin-D within the CDC27-silenced cells, similar to the *iglC* mutant in untreated cells (Fig. 5B and C). Thus, the CDC27 ubiquitin ligase is crucial for the ability of the WT strain to limit fusion of the FCP to lysosomes. Taken together, our data indicate that the CDC27 ubiquitin ligase is essential for *F. tularensis* to evade lysosomal fusion, while the USP22 ubiquitin-specific peptidase and the PI4KCA PI4 kinase are only required for bacterial proliferation in the cytosol, but play no detectable role in evasion of lysosomal fusion.

To determine whether the three host factors were involved in interaction with autophagy during late stages of infection, similar studies were conducted at 12 h and 24 h post infection. The results showed that at 12–24 h of infection the % of co-localization of the WT FCPs with both LAMP-2 and cathepsin-D positive compartments did not change from the 2 h time point in the CDC27, USP22, or PI4KCA RNAi-treated cells. Thus, in contrast to mouse-derived cells, late stages of intracellular replication of *F. tularensis* within the cytosol of human-derived cells do not involve re-entry to the endosomal-lysosomal pathway through autophagy. These data show that interaction of cytosolic *F. tularensis* with autophagy during late stages of proliferation within the cytosol is specific to the mouse model but does not apply to the human host.

### The role of CDC27, USP22 and PI4KCA in phagosomal escape

**Figure 6:**
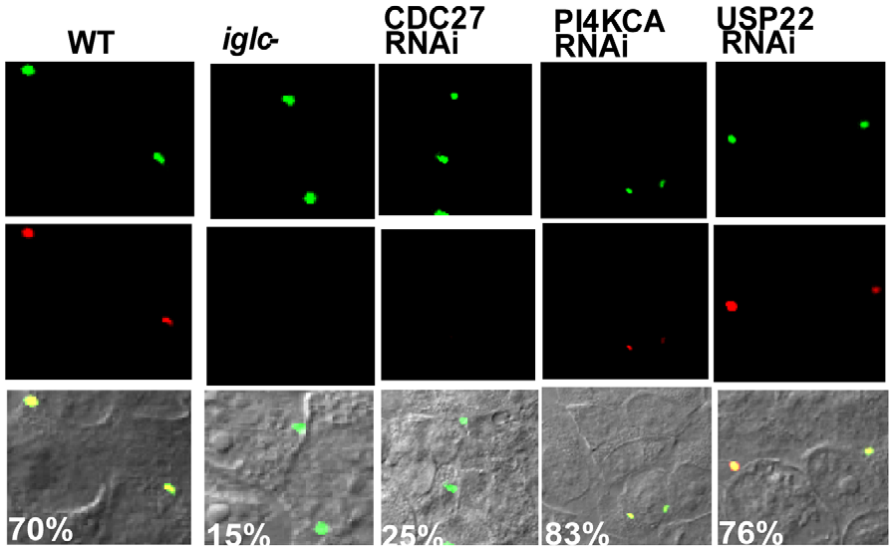
CDC27 but not USP22 or PI4KCA is required for bacterial escape into the cytosol. Representative confocal microscopy images of the WT *F. tularensis* within untreated or RNAi-treated cells to determine phagosomal escape. Phagosomal escape was determined by the ability of GFP-expressing intracellular bacteria to bind anti-*F. tularensis* antibody (red) loaded into the host cell cytosol after preferential permeabilization of the plasma membrane, compared to the bacteria found within intact vacuoles (green) that are impermeable to the antibody. The % of cytosolic bacteria are shown, based on examination of at least 100 bacteria at 2 h after infection. The results are representative of three independent experiments performed in triplicate.

To determine the role of the USP22, CDC27 and PI4KCA factors in phagosomal escape directly, we utilized a fluorescence microscopy-based phagosomal integrity assay, to differentially label bacteria that are cytosolic or within a compromised phagosome vs. bacteria enclosed within an intact phagosome, as we described previously (see Experimental Procedures). This is achieved by loading the host cell cytosol with anti-*F. tularensis* antibody after preferential permeabilization of the plasma membrane. Consistently, ∼85% of the *iglC* mutant control remained in intact phagosomes in untreated cells (Fig. 6). In untreated HEK293T cells infected by the WT strain for 2 h, ∼70% of the bacteria were cytosolic or within disrupted FCPs (Fig. 6). Similarly, ∼80% of the intracellular bacteria were cytosolic in USP22 and PI4KCA RNAi-silenced cells. In contrast, only 25% of the WT strain escaped into the cytosol within the CDC27-silenced cells. The data are consistent with our studies above on phagosome biogenesis in the RNAi-treated cells (Fig. 5). Therefore, both PI4KCA and USP22 are not required for phagosomal escape but are essential for bacterial replication within the cytosol, while CDC27 is essential for phagosomal escape of *F. tularensis* into the host cell cytosol.

## Discussion

Previously, we have shown that intracellular trafficking and cytosolic proliferation of *F. tularensis* within *D. melanogaster* derived-cells is similar to that of mammalian macrophages at the cellular level [1]. However, there are some clear differences in the bacterial factors involved in these processes in *D. melanogaster* derived-cells compared to human macrophages [22], [23]. At least 186 arthropod host genes have been confirmed to be required for intracellular proliferation of *F. tularensis* with *D. melanogaster*-derived cells. Due to the high throughput nature of our screen and automated measurement of intensity of the GFP fluorescence, our screen does not differentiate mutants defective in intracellular replication from the ones that are severely defective in entry into S2 cells. Importantly, three mammalian homologue genes have been confirmed to play an essential role in modulation of phagosome biogenesis, phagosomal escape and proliferation of *F. tularensis* within the cytosol of human cells. Although we only selected 12 mammalian homologues, our data clearly indicate that there are major differences between the mammalian and arthropod host factors required for intracellular proliferation of *F. tularensis*. This is consistent with our recent findings that there are some clear differences in the bacterial factors involved in these processes in *D. melanogaster* derived-cells compared to human macrophages [22], [23]. This is despite the clear presence of some similar mechanisms involved in proliferation in both host cells through modulation of conserved eukaryotic factors.

Some identified host factors are relevant to previously published host factors and pathways that are manipulated by *F. tularensis* and other intracellular pathogens [26], [29], [30], [35], [36], [37]. Consistent with other findings, we identified some host factors involved in actin or microtubule rearrangement that are required for invasion by *F. tularensis* [13], [37], [38]. Our data show that an RNAi knockdown in Pi3k21B results in enhanced growth of *F. tularensis,* consistent with the negative regulation of this gene during infection by *F. tularensis* [37]. It is also noteworthy that the CD36 family scavenger receptor (Pes) RNAi target found to be required for uptake of *L. monocytogenes* and *Mycobacterium* [26], [30] is also required for infection by *F. tularensis*, suggesting that *F. tularensis* may use this receptor to enter the host cell.

Within the host cell, *F. tularensis* resides transiently within a late endosome-like phagosome that becomes acidified upon acquisition of the vATPase proton pump with limited fusion to the lysosomes, followed by bacterial escape into the cytosol [1]. We identified novel host factors that are likely involved in modulation of biogenesis of the FCP such as VhaAC39, a component of the vATPase pump, or the Rab proteins such as Rab8 and Rab10 that are involved in vesicular trafficking. Consistent with the findings that iron is a nutritional requirement for intra-macrophage growth by *F. tularensis* [39], [40], [41], we identified host factors involved in iron binding and transport processes, such as *cyp317a1*, and *tsf1*, to be required for intracellular proliferation of *F. tularensis*.

To decipher whether some of the mechanisms of pathogenesis deployed by this intracellular pathogen within arthropod and mammalian hosts are similar, we selected 12 human gene homologues that suppress intracellular replication within *Drosophila* cells and these genes belong to different functional categories. We show that silencing of CDC27, USP22 and PI4KCA suppressed intracellular replication of *F. tularensis* within mammalian cells. The USP22 ubiquitin-specific peptidase belongs to a large family of proteins that contain a carboxy terminal ubiquitin hydrolase [42], [43]. Furthermore, endogenous USP22 contributes a deubiquitylating activity to the human SAGA complex, required for the function of transcription activators in eukaryotes [43]. This activity is directed at the core histone H2B, where USP22 may regulate transcription via alterations in levels of histone ubiquitylation [43]. Silencing of USP22 does not affect evasion of lysosomal fusion or phagosomal escape by *F. tularensis*, but suppress replication of *F. tularensis* within the cytosol of mammalian cells. These data clearly show that *F. tularensis* modulate host cell cytosolic factors to enable bacterial proliferation within the cytosol. It is likely that the ubiquitin hydrolase activity of USP22 contributes to permissiveness of the host cell cytosol for bacterial proliferation.

Silencing of PIK4ca or PI4K-III-α, which encodes a type III PI4-kinase α subunit, inhibits intracellular replication of *F. tularensis* between 8 and 24 hours after infection but not at the early time points. There are 4 known cellular PI4-kinases involved in the phosphorylation of PI to yield PI4P. Their primary distinction is their localization within the cell, resulting in different biological outcomes [44]. Interestingly, silencing of PI4K-III-α significantly reduces hepatitis C virus (HCV) RNA replication [45]. In contrast to *F. tularensis, L. monocytogenes* utilizes PI4K II-α and PI4K II-β to modulate its entry into human cells, but does not require PI4K-III-α for phagocytosis [46], which might highlight alternate mechanisms these two bacteria utilize to manipulate phosphoinositide metabolism. Silencing of PI4K-III-α did not alter evasion of lysosomal fusion and phagosomal escape by *F. tularensis*. Therefore, PI4KCA is not required for evasion of lysosomal fusion, but it is essential for bacterial proliferation in the host cytosol, where many transduction pathways downstream of PI4KCA may favor intracellular replication of *F. tularensis*. The differential and temporal requirement of PI4KCA during infection by *F. tularensis* suggests modulation of PI4KCA-dependent signal transduction pathways and modulation of phosphoinositide metabolism by *F. tularensis* are triggered after phagosomal escape and initiation of bacterial proliferation within the cytosol.

The CDC27, or APC3 protein, is an essential member of the anaphase-promoting complex (APC), an E3 ubiquitin ligase in the ubiquitin-mediated proteolysis pathway involved in regulation of the cell cycle [47]. Our data show that silencing of CDC27 enhances fusion of the FCP to the lysosomal vesicles and restricts phagosomal escape. The E3 ubiquitin ligase may be essential to recruit bacterial or host substrates for polyubiquitination. The role of the CDC27 E3 ubiquitin ligase in modulation of biogenesis of the FCP and the role of the USP22 ubiquitin-specific peptidase in bacterial proliferation within the cytosol suggests that *F. tularensis* may utilize or exploit host ubiquitin turnover in distinct mechanisms during the phagosomal and cytosolic phases of the bacteria.

In mouse bone marrow-derived macrophages (BMM), *F. tularensis* re-enter the endocytic compartment within ∼20 hrs of infection, via an autophagy-like process [10]. Our data show that *F. tularensis* does not re-enter the endosomal-lysosomal pathway during late stages of proliferation within the cytosol of human cells. Therefore, interaction of *F. tularensis* with autophagy is specific to the murine model but does not apply to the human host. This is likely due to differences in the innate immunity between the two host species. Our data caution extrapolation of findings about *F. tularensis* within the murine model to human infection.

Taken together, we have utilized the arthropod-vector model of *F. tularensis*, *D. melanogaster*, in a genome-wide RNAi-based forward genetic screen to identify host factors that affect intracellular proliferation of *F. tularensis,* and have identified some conserved mammalian homologues required for modulation of phagosome biogenesis, phagosomal escape and proliferation of *F. tularensis* within the cytosol. Unique as well as conserved host factors are exploited by *F. tularensis* to proliferate within arthropod and mammalian-derived cells. This is consistent with the differences in the molecular mechanisms utilized by *F. tularensis* to evade lysosomal fusion and escape into the cytosol within human macrophages and arthropod-derived cells [22], [23]. Our data indicate that *F. tularensis* utilizes host ubiquitin turnover and phosphoinositide metabolism in distinct mechanisms during the phagosomal and cytosolic phases of the bacteria. Our data will facilitate deciphering the ecology, and patho-adaptation of *F. tularensis* to the arthropod vector and its role in bacterial ecology and patho-evolution to infect mammalian cells.

## Materials and Methods

### Bacteria, media and tissue cultures

*Francisella tularensis ssp*. *novicida* strain U112 and its isogenic mutants harboring the plasmid pKK214, which encodes *gfp*, were used in all experiments and have been described elsewhere [34], [48], [49]. Bacteria were cultured on tryptic soy agar (TSA) plates supplemented with 0.1% cysteine and 10 mg ml^−1^ tetracycline as required. *Drosophila* S2R+ cells and dsRNA used for the primary and secondary RNAi screens were supplied by the *Drosophila* RNAi Screening center (Harvard Medical School, (http://flyrnai.org/). *Drosophila* cells were maintained at 24°C in tissue culture flasks containing Schneider's *Drosophila* medium (Gibco) supplemented with 10% heat inactivated fetal bovine serum (SDM-10).

### High throughput *D. melanogaster* genome-wide RNAi screen

*F. tularensis* infections of S2R+ cells for the primary and secondary RNAi screens were performed in 384-well plates. S2R+ cells were re-suspended in serum-free Schneider's *Drosophila* medium (SDM), and ∼3.0×10^4^ S2R+ cells in 10 µl were added to each well of the 384-well plates containing RNAi targets using a WellMate Microplate dispenser (Thermo Scientific). After 1 hr at RT, 30 µl of SDM-10 were added to each well, and then cells were incubated at 25°C for 4 days to allow for knockdown of target transcripts and protein degradation. The S2R+ cells were then infected by adding 10 µl of a suspension of GFP-expressing *F. tularensis ssp*. *novicida* strain U112 or its isogenic mutants at ∼MOI 20 in SDM-10 using a WellMate Microplate dispenser. The S2R+ cells were incubated at 28°C for 2 hrs, washed once, and 50 µg/ml gentamicin in SDM-10 was added to kill extracellular bacteria. The media was removed, and 50 ul of fresh SDM-10 was added, then cell were incubated at 28°C. Four days post infection, bacterial proliferation was quantified by reading GFP fluorescence using the Analyst GT plate reader (Molecular Devices) and filter sets 485 nm excitation, 530 nm emission and 505 nm dichroic mirror.

Overall for the genome-wide primary screen and secondary quantitative screen, at least 3 independent experiments were performed, each RNAi target was tested in duplicate or in multiple wells, and the Z-scores of fluorescence intensities within each 384 well plate were calculated using bioinformatics tools provided by the *Drosophila* RNAi Screening center (Harvard Medical School, http://flyrnai.org/). Raw data of *F. tularensis* GFP fluorescence intensity generated by the plate reader in each plate was transformed into z-scores in the form of (Raw Data-Plate median)/interquartile Range heat map using the *Drosophila* RNAi screening center website (http://flyrnai.org/) analysis tool to select RNAi targets that suppressed intracellular bacterial proliferation. In addition, wells suspected to have edge effects (Yellow clusters in the Z-score heat map, Fig. 1A) or dispensing errors were not considered as hits. The following host factors not found in our primary screen were tested again in the secondary screen: Rab11, Rab35, Rab7, Arf102F, CG3523, tor, ckiibeta, vhappa1-1, vhaAC39, CG5691, CG11814, arc-p20, act5c, act57b, rab1, rab10, rab2, rab21, rab5, rab8, crq (CD36), CG7228 (CD36 family), and CG2699 (Pi3K21B). Student's *t*-test was used and the *P*-value was obtained. GraphPad Prism 5 was used for statistical analyses.

### Immunofluorescence and automated fluorescence microscopy of the screen

After 2 hr of infection of S2R+ cells, the media was removed; 500 µg/ml gentamicin in SDM-10 was added to kill extracellular bacteria. Twenty-four hours post-infection, the 384-well plates were centrifuged, the medium was aspirated using a 24 channel wand (V&P scientific, inc) and samples were fixed by adding 50 µl of 3.7% paraformaldehyde in PBS per well and incubating for 30 min at RT. Wells were washed with PBS, permeabilized with 0.1% Triton X-100, blocked with 3% BSA, then bacteria were labeled with 1∶4000 anti-*F. tularensis* antibodies, followed by secondary labeling with 1∶4000 alexa fluor 555-coupled donkey anti-goat antibodies. The S2R+ cells were labeled with 5 µg/ml Hoechst dye in 3% BSA. Samples were then washed with PBS and kept in PBS at 4°C until automated fluorescence microscopy analysis was performed.

Fluorescence images of infected S2R+ cells in 384- well plates were acquired using the ImageXpress Micro automated epifluorescent microscope (Molecular devices) equipped with the Photometrics CoolSNAP ES digital CCD camera, and the MetaXpress cellular image analysis software. Images were acquired with a 40X magnification, Plan Fluor ELWD lens, DAPI and CY3 filters. Images from the stained nuclei and antibody labeled bacteria were collected at five random fields within each well, and further analyzed using image J NIH software; l images within each channel were set with the same parameters.

### Silencing of host factors in HEK 293T cells

HEK293T cells were maintained in Dulbecco's modified Eagle's medium (DMEM) (Invitrogen, Carlsbad, CA) supplemented with 10% heat inactivated fetal bovine serum (FBS). One day before transfection, 5×10^4^ cells/ml were seeded in six-well plates and on glass cover slipsin 24-well plates. The RNAi clones were purchased from Santa Cruz (Santa Cruz, CA.), and transfected as recommended by the manufacturers. Knockdown of RNAi silenced genes was evaluated by western blot probed with anti-PI4KCA from Proteintech group Inc, (Chicago, IL), anti- CDC27 or anti-USP22 antibodies from Abcam (Cambridge, MA). Anti-actin anti-serum was obtained from Sigma (St. Louis, MO) and was used to re-probe the Western blots for a loading control.

### Confocal laser scanning microscopy

Untreated HEK293T cells and successfully silenced cells were infected with the *F. tularensis* subsp. *novicida* for 1 h using MOI of 10. After infection, the cells were washed three times, and treated with 50 µg/ml gentamicin for 1 hr to kill extracellular bacteria. The cells were processed for confocal microscopy as we described previously [12], [34]. Bacteria were labeled with anti*-F. tularensis* antibody (1∶4000) and appropriate secondary antibodies (Molecular Probes Invitrogen, Carlsbad, CA). The anti-LAMP-2 (H4B4) 1∶2000 monoclonal antibody (developed by J. T. August and J. E. K. Hildreth) was obtained from the Developmental Studies Hybridoma Bank of University of Iowa, and anti-cathepsin D mAb (1∶200) was obtained from BD transduction (San Jose, CA). The images were captured using the Fluoview FV-1000 confocal microscope, and are presented in the figures as a single z section.

### Quantitation of vacuolar and cytoplasmic *F. tularensis* within semi-permeabilized HEK293T cells

To evaluate the proportions of cytoplasmic and vacuolar *F. tularensis*, untreated or siRNA treated HEK293T cells in 24-well plates (5×10^4^ per well) were infected with the WT strain or *iglC* mutant. Two hours after infection, cells were washed once with KHM buffer (110 mM potassium acetate/20 mM Hepes/2 mM MgCl_2_, pH 7.3), and their plasma membranes were selectively permeabilized with 35 µg/ml digitonin and 1∶200 goat anti-*F. tularensis* antibody, in DMEM medium, for 15 min at room temperature. After washing once with KHM buffer, goat anti-*Francisella* antibody was added for 30 more min and incubated at 37°C to label cytoplasmic bacteria. After washing in KHM buffer, cells were fixed and permeabilzed with ice cold methanol for 5 min at −20°C and blocked with 3% BSA for 1 h at room temperature. Both vacuolar and cytoplasmic bacteria were labeled with 1∶200 mouse anti-*F. tularensis* mAb for 1 h at room temperature. Secondary donkey anti-mouse conjugated to Alexa-Fluor488 and donkey anti-goat conjugated to Alexa-Fluor555 were obtained from Molecular Probes Invitrogen (Carlsbad, CA), were added for 1 h. Coverslips were mounted and cells were examined by confocal microscopy. The images were captured using the Fluoview FV-1000 confocal microscope, and are presented in the figures as a single z section.

### Statistical Analysis

Student's *t*-test was used and the *P*-value was obtained. GraphPad Prism 5 was used for statistical analyses. *P*-value <0.05 was considered statistically significant.

## Supporting Information

Table S1 Confirmed host factors required for proliferation of *F. tularensis* in *D. melanogaster* cells. Further descriptive information on individual RNAi target can be found at the Drosophila RNAi Screening Center (DRSC) website (flyrnai.org) using the indicated DRSC amplicon. L (*L. monocytogenes*), M (*Mycobacterium*), C (*Chlamydia*). Phenotype strength are described as strong (3), medium (2), or weak (1). * Selected genes homologues in mammalian cells.
(0.12 MB XLS)
